# Scaling Up Tuberculosis Preventive Treatment in the Brazilian Amazon (2016-2024): a Programmatic Report on Multi-sector Integration in Surveillance, Health Services and Academia

**DOI:** 10.1590/0037-8682-0570-2025

**Published:** 2026-07-17

**Authors:** Jair dos Santos Pinheiro, Lara Bezerra de Oliveira Assis, Djane Clarys Baia-da-Silva, Vitória Celestino Oliveira, Leandro Sousa Garcia, Renata Spener-Gomes, Amanda França Silva Aguiar, Andresa Carraro Rocha, Alexandra Brito Souza, Allyson Guimarães Costa, Amélia Nunes Sicsú, Flávia Regina Souza Ramos, Bruno de Bezerril Andrade, Marcelo Cordeiro-Santos

**Affiliations:** 1 Universidade do Estado do Amazonas, Manaus, AM, Brasil.; 2 Fundação de Medicina Tropical Dr. Heitor Vieira Dourado, Manaus, AM, Brasil.; 3 Fundação de Vigilância em Saúde Dra. Rosemary Costa Pinto, Manaus, AM, Brasil.; 4 Secretaria Municipal de Saúde de Manaus, Manaus, AM, Brasil.; 5 Universidade Federal de Santa Catarina, Florianópolis, SC, Brasil.; 6 Fundação Oswaldo Cruz Amazônia, Instituto Leônidas e Maria Deane, Manaus, AM, Brasil.; 7 Universidade Nilton Lins, Manaus, AM, Brasil.; 8 Universidade Federal do Amazonas, Manaus, AM, Brasil.; 9 Fundação Hospitalar de Hematologia e Hemoterapia do Amazonas, Manaus, AM, Brasil.; 10 Universidade do Estado de Santa Catarina, Florianópolis, SC, Brasil.; 11 Johns Hopkins University, School of Medicine, Division of Infectious Diseases, Baltimore, Maryland, USA.; 12 Instituto MONSTER de Ensino, Assistência, Pesquisa e Desenvolvimento Tecnológico em Saúde, Salvador, BA, Brasil.; 13 Instituto de Pesquisa Clínica e Translacional, Medicina Zarns, Clariens Educação, Salvador, BA, Brasil.; 14 Fundação Oswaldo Cruz, Instituto Gonçalo Moniz, Laboratório de Pesquisa Clínica e Translacional, Salvador, BA, Brasil.

**Keywords:** Tuberculosis, Latent tuberculosis, Implementation science, Medically underserved area, South America, Brazil

## Abstract

**Background:**

Tuberculosis (TB) remains a major public health challenge in Brazil, particularly in the state of Amazonas, where geographic, socioeconomic, and health system barriers hinder the implementation of TB preventive treatment (TPT). This study describes the scale-up of TPT, supported by integrated actions across surveillance, health services, and academia in this high-burden setting.

**Methods:**

We conducted a retrospective implementation analysis of TPT in Amazonas, Brazil (2016-2024), using data from the IL-TB system, SINAN-TB, and HIV/AIDS Clinical Monitoring Panel. A structured set of indicators was used to assess TPT delivery, decentralization, diagnostic practices, regimen uptake, and treatment outcomes. Proportions and rates were estimated with 95% confidence intervals (95% CI).

**Results:**

A total of 8,120 individuals initiated TPT, including 5,001 TB contacts, whereas more than 70,000 contacts were recorded in the SINAN-TB. Following the IL-TB implementation in 2019, the indicators showed a sustained expansion of TPT delivery. By 2024, TPT initiation among contacts reached 18.4% (95% CI: 17.6-19.3), and 959 PLHIV initiated TPT, corresponding to 441.33 per 1,000 antiretroviral therapy. The proportion of TPT initiation in primary health care increased from 19.3% (95% CI: 13.8-26.4) in 2018 to 55.2% (95% CI: 53.3-57.1) in 2024. Use of 3HP (weekly rifapentine plus isoniazid for three months) rose from 12.7% (95% CI: 10.6-15.1) in 2021 to 93.3% (95% CI: 92.3-94.2) in 2024. Completion reached 84.8% (95% CI: 82.9-86.6) and remained above 80% thereafter.

**Conclusions:**

TPT scale-up in Amazonas was accompanied by expanded access, greater decentralization, and rapid adoption of shortened regimens, although important coverage gaps among priority populations remain.

## INTRODUCTION

Tuberculosis (TB) remains a major public health challenge worldwide. In 2023, the World Health Organization (WHO) estimated 10.8 million incident cases and 1.25 million deaths, highlighting its continued global impact. In Brazil, TB is a leading cause of morbidity and mortality, with over 84,000 cases reported in 2024, while 6,025 deaths were attributed to the disease in 2023^1^ ^,^ ^2^.

Building on the national context, the state of Amazonas in Brazil represents a high-burden setting, with more than 4,000 new TB cases reported annually and incidence rates exceeding 70 per 100,000 inhabitants. Mortality has also increased in recent years, surpassing 250 deaths annually. Time-series data show a clear increase when comparing pre-pandemic averages (approximately 3,000-3,200 cases and 150-170 deaths annually in 2017-2019) with post-pandemic levels (over 4,000 cases and 215-280 deaths annually from 2022 onwards), reflecting disruptions in TB services^3^. The region’s unique social and geographic characteristics, including widespread poverty, limited access to healthcare, and large indigenous and riverine populations^4^, have made TB control particularly complex. Vulnerable groups, such as people living with HIV (PLHIV), household contacts, Indigenous people, and residents of remote riverine communities, are disproportionately affected, facing obstacles in diagnosis, care, and the uptake of preventive strategies^1^ ^,^ ^2^ ^,^ ^5^ ^-^ ^8^.

Regarding latent tuberculosis infection (LTBI), approximately one-quarter of the global population is infected with *Mycobacterium tuberculosis*. A systematic review estimated a global prevalence of 24.8% (95% CI: 19.7-30.0) using interferon-gamma release assays (IGRA) and 21.2% (95% CI: 17.9-24.4) using the tuberculin skin test (TST). In Brazil, the prevalence of LTBI is estimated to be approximately 20%^9^.

Brazil has made significant advances in LTBI and TB preventive treatment (TPT), with Manaus, the capital of the state of Amazonas, emerging as a regional research and innovation hub. Studies have identified key barriers and facilitators of LTBI care and informed strategies to expand preventive therapy, particularly among PLHIV. Some early work elucidated the factors driving losses in the LTBI care cascade and underscored the need for patient-centered, culturally sensitive approaches in high-burden settings^5^ ^,^ ^7^. Subsequent surveys have highlighted the importance of training frontline healthcare providers and engaging at-risk contacts in care^10^ ^-^ ^13^. Research involving PLHIV has contextualized and optimized novel strategies for LTBI diagnosis and treatment, revealing critical operational challenges and opportunities for scaling up these programs^14^.

Notably, the participation of the state of Amazonas in nationwide research consortia, such as the Regional Prospective Observational Research for Tuberculosis (RePORT-Brazil), is epidemiologically representative, and has provided a unique lens into the regional disease burden and intervention effectiveness^15^. These multicenter cohort studies have enabled robust evaluations of clinical outcomes, management and implementation of new diagnostics and regimens, including IGRA and short-course preventive therapies^6^ ^,^ ^16^ ^-^ ^21^.

Despite policy efforts and scientific advances, the translation of TPT guidelines into routine care remains slow. Persistent barriers include logistical challenges, knowledge gaps among healthcare providers and patients, limited diagnostic resources, and variability in programmatic engagement^22^. Scaling up innovative shorter TPT regimens and diagnostic modalities requires requires leadership, investment and adaptive, evidence-driven strategies suited to local realities^23^ ^-^ ^26^.

In this study, we aimed to describe the evolution and programmatic impact of TPT scale-up in the state of Amazonas from 2016 to 2024. We examined trends in TPT initiation and completion, the effectiveness of implementation models, and the integration of research findings into public health decision-making. Ultimately, this study seeks to identify key operational enablers and persistent challenges to guide future strategies for TB elimination, leveraging both local and national scientific contributions.

## METHODS

### Study design and setting

This study employed a retrospective real-world implementation analysis design. It evaluated the scale-up and performance of TPT delivery in the state of Amazonas, Brazil, from January 2016 to December 2024. Our setting was selected because of its persistently high TB burden and the strategic implementation of integrated public health interventions to meet the WHO End TB Strategy targets. Many of the integrated strategies and innovations adopted in the region were informed by operational research and collaborative studies led by the Fundação de Medicina Tropical Dr. Heitor Vieira Dourado in Manaus, Brazil (FMT-HVD). These studies provided key evidence regarding barriers, facilitators and solutions for preventive TB care in the Amazon and other settings in Brazil^5^ ^,^ ^6^ ^,^ ^14^ ^,^ ^18^ ^,^ ^20^ ^,^ ^21^.

Data sources and variables

Programmatic and epidemiological data were obtained from routine public health information systems maintained by the Fundação de Vigilância em Saúde do Amazonas Dr. Rosemary Costa Pinto (FVS-RCP) and the Ministry of Health, Brazil. The data were updated in October 2025.

**Information System for notification of people undergoing treatment for LTBI (IL-TB System):** The primary data source for assessing TPT initiation, prescribed regimens, site of care (primary health care [PHC] or specialized services), and treatment outcomes.

**Notifiable Diseases Information System (SINAN-TB):** Used to identify new TB notifications and determine the number of eligible contacts recorded.

**HIV/AIDS Clinical Monitoring Indicator Panel (PIMC HIV/AIDS):** Used to obtain the total number of PLHIV initiating Antiretroviral Therapy (ART) and those currently on ART in the state (annual stock), used as the denominator for TPT rate among PLHIV.

**Administrative Data:** This includes documentation of key programmatic developments­, such as the introduction of new regimens and diagnostic tools within the scope of Coordenação Geral de Tuberculose e Micoses Endêmicas, Ministério da Saúde, Brazil (CGTM/MS), along with management reports and records of activities from the Amazonas State Tuberculosis Programme, as evidence of integrated actions between surveillance, health services and academia.

These multiple datasets were integrated and analyzed using models and recommendations developed by local and multicenter research, particularly those evaluating representativeness, diagnostic innovation, and losses along the TB preventive care cascade^5^ ^,^ ^7^ ^,^ ^14^.

The IL-TB system was developed and implemented nationally in 2019 by the CGTM/MS as a web-based platform and subsequently decentralized to support state and municipal coordination of tuberculosis programs and healthcare facilities. It includes numerous automated data quality and consistency checks, such as flagging potential duplicity and incorrect registration of a TPT-eligible group or treatment regimen. These functionalities are available to all system users for auditing and validating data quality in real time^24^.

The SINAN-TB system is the official national surveillance database for notifiable diseases in Brazil, including tuberculosis, and comprises standardized notification and follow-up forms that capture demographic, clinical, laboratory, and operational variables^27^.

The HIV/AIDS Clinical Monitoring Panel (PIMC) is a national tool developed by the Ministry of Health that integrates data from multiple surveillance and HIV care management information systems into a unified database in Brazil^28^. It provides aggregated indicators for individuals who initiated and are currently receiving ART, enabling the construction of denominators for TPT indicators among PLHIV.

### Statistical analysis, indicators and definitions

The implementation analysis was based on a structured set of 10 indicators covering access, implementation, decentralization, adherence and diagnostic processes, aligned with the CGTM/MS^29^ and WHO frameworks, along with additional indicators adapted and developed to better capture programmatic performance in the Amazonas context. A detailed description of all indicators, including definitions, operationalization, data sources and limitations, is provided in the Supplementary Material Table 1 **.**

For each indicator, the numerators and denominators were defined according to the measure and data source. Proportions and rates were derived from routine surveillance data, with population-based indicators using annual denominators, such as ART stock. Estimates were presented with 95% confidence intervals (95% CI), using Wilson’s method for proportions and exact Poisson method for rates. Absolute differences were expressed in percentage points, and time-to-implementation was summarized using the median and interquartile ranges.

Given the use of aggregated data from multiple information systems without individual-level linkage, this study adopted an ecological analytical approach, combining descriptive trend analysis with structured numerator-denominator indicators to assess programmatic performance across municipalities and over time. For indicators related to contacts and PLHIV, a six-month moving average was calculated to assess the temporal patterns of TPT implementation.

The analysis employed approaches tested and recommended in prior operational studies conducted in the state of Amazonas and the broader RePORT-Brazil network^30^, using R software.

### Ethics approval, consent to participate, and data governance

According to Resolution No. 510 of April 7, 2016, of the National Health Council of Brazil, which governs the ethical standards for research in the Human and Social Sciences in Brazil, the need for informed consent may be waived in cases of the use of anonymized secondary data if it is justified and approved by the Research Ethics Committee. Our study protocol was approved by the Ethics Committee of the FMT-HVD (CAAE: 74993423.8.0000.0005).

Data extraction, processing and anonymization of the IL-TB and SINAN-TB databases were performed by the technical staff of the State Tuberculosis Control Programme (FVS-RCP) as part of routine public health surveillance activities. Data from the HIV/AIDS Clinical Monitoring Panel (PIMC) are publicly available and periodically released by the Brazilian Ministry of Health without individual identification. The authors external to the Amazonas State Tuberculosis Control Program did not have access to the individual-level data.

## RESULTS

Over the study period (2016-2024), 8,120 new TPT cases were recorded in the IL-TB system, of which 5,001 (61.6%) were contacts of TB cases, while more than 70,000 contacts were recorded in the SINAN-TB system. Following the implementation of the IL-TB in 2019, a set of measurable and operational indicators was defined to assess LTBI surveillance and the cascade of care. The proportion of contacts initiating TPT increased from <5% in earlier years to 18.4% in 2024 (95% CI: 17.6-19.3). Among PLHIV, with approximately 2,000 ART initiations annually, 959 individuals initiated TPT in 2024, corresponding to 441.33 per 1,000 ART initiators. The proportion of TPT initiation in PHC increased from 19.3% (95% CI: 13.8-26.4) in 2018 to 55.2% (95% CI: 53.3-57.1) in 2024, while the absolute difference in completion between levels of care ranged from +14.7 to −2.9 percentage points over time (Supplementary Material Table 2).

The diagnostic and treatment indicators varied across the study period. IGRA utilization remained around 10-13% of initiators, whereas TST reached 92.8% (95% CI: 90.2-94.7) in 2020 and 73.0% (95% CI: 71.3-74.7) in 2024. The proportion of TPT initiations using weekly rifapentine plus isoniazid for three months (3HP) increased from 12.7% (95% CI: 10.6-15.1) in 2021 to 93.3% (95% CI: 92.3-94.2) in 2024, with a median time to local implementation of 12 months (IQR: 8-28.5). Completion rates reached 84.8% (95% CI: 82.9-86.6) in 2022 and remained above 80% in subsequent years, with consistently higher values observed for shortened regimens than long-duration regimens (Supplementary Material Table 2). 

### Timeline and programmatic developments

**FIGURE 1:**
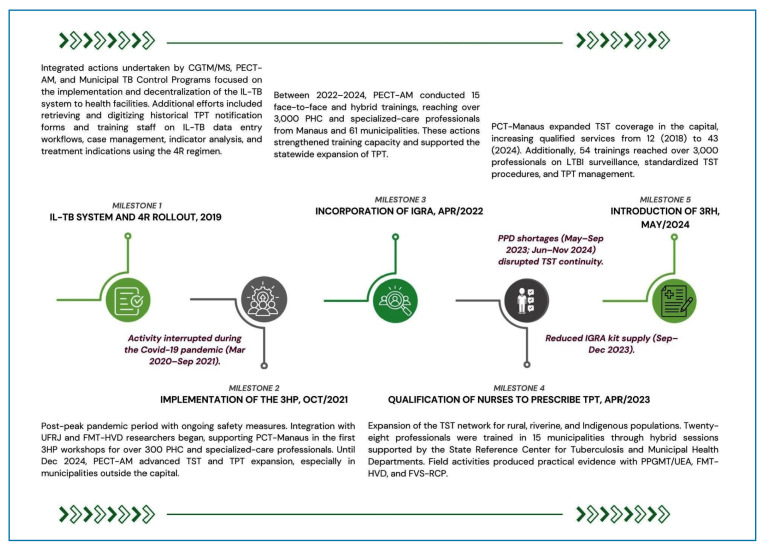
Timeline of key TPT implementation developments in Brazil, actions and results of the integration of the public health service and academia to expand access to TPT in Amazonas, Brazil, 2019-2024. **Notes: CGTM/MS:** Coordenação Geral de Tuberculose e Micoses Endêmicas/Ministério da Saúde, Federal District, Brazil; **PECT-AM:** Programa Estadual de Controle da Tuberculose, Amazonas, Brazil; **IL-TB:** Information System for notification of people undergoing treatment for LTBI; TPT, TB Preventive Treatment; **4R:** daily doses of rifampicin for four months; **UFRJ:** Universidade Federal do Rio de Janeiro, Rio de Janeiro, Brazil; **UEA:** Universidade do Estado do Amazonas, Amazonas, Brazil; **FMT-HVD:** Fundação de Medicina Tropical Dr. Heitor Vieira Dourado, Amazonas, Brazil; **PCT-Manaus:** Programa de Controle da Tuberculose, Manaus, Amazonas, Brazil; **3HP:** weekly doses of rifapentine plus isoniazid for three months; **PHC:** primary health care; **TST:** tuberculin skin test; **IGRA:** interferon-gamma release assay; **PPD:** purified protein derivative; **3RH:** daily doses of rifampicin plus isoniazid for three months. **Source:** PECT-AM/FVS-RCP, October 2025.

The introduction of the IL-TB system and rollout of the 4R regimen (4 months of daily rifampicin) in 2019 marked the beginning of large-scale electronic notification and decentralized TPT management. Rapid subsequent advances included the COVID-19-induced operational adaptation (March 2020-September 2021) and the implementation of 3HP in October 2021 through collaborative workshops involving more than 300 PHC and specialized services professionals in the state capital, Manaus. The integration of IGRA testing in April 2022, qualification of nurses for TPT prescription in April 2023, and incorporation of the 3RH regimen (3 months of daily Rifampicin (R) plus Isoniazid (H)) in May 2024 further diversified and strengthened the regional response. These developments were accompanied by intermittent shortages of both IGRA kits and purified protein derivatives (PPD), requiring operational flexibility and intersectoral coordination. The strategic integration of public health service management and academic partnerships ensured ongoing workforce education of the workforce and sustained expansion to access to treatment, especially for priority groups in remote areas and among vulnerable populations (Figure 1).

### Spatial expansion and implementation of 3HP

**FIGURE 2:**
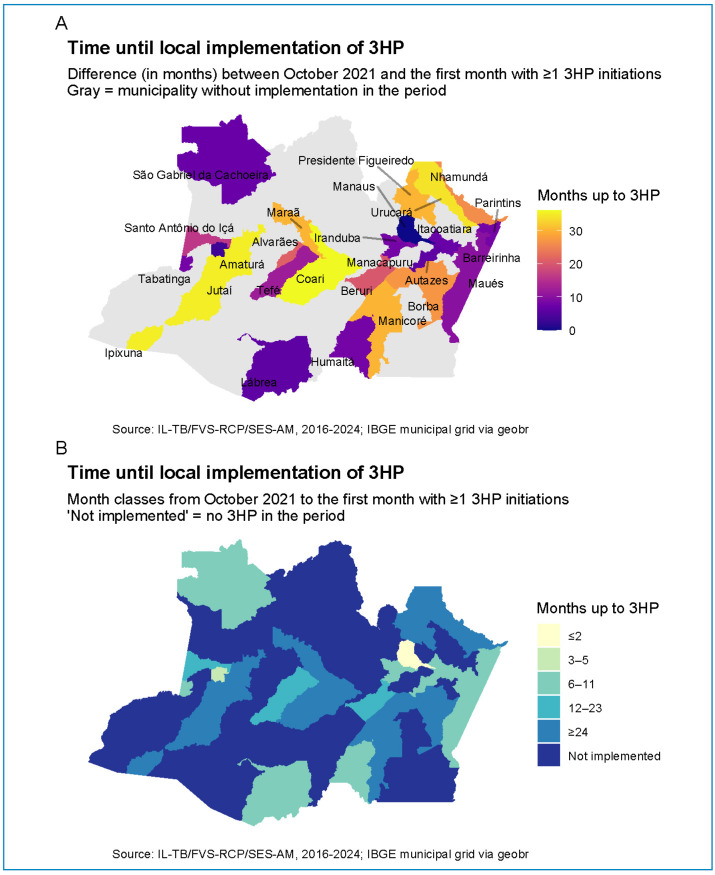
Time until implementation of the 3HP regime according to the municipality of the health service that initiated the TPT in Amazonas, Brazil, Oct 2021-Dec 2024 (N=5,113). **Note:** 3HP, weekly doses of rifapentine plus isoniazid for three months. **Source:** Information System for notification of people undergoing treatment for LTBI (IL-TB), Amazonas, Brazil, October 2025.

Central hubs, including Manaus and its surrounding areas, achieved rapid uptake, implementing 3HP within 5 months of its official release. In contrast, peripheral and geographically isolated municipalities, such as São Gabriel da Cachoeira and Tabatinga, experienced significant delays-some exceeding 24 months while others did not implement 3HP at all during the evaluation period. Overall, more than two-thirds of municipalities completed the introduction of 3HP by December 2024, but nearly one-third lagged behind, highlighting persistent disparities and barriers due to infrastructure limitations, logistical challenges and limited programmatic resources. Early implementation clustered around regions with higher health system capacity and better access to research support, underscoring the value of targeted interventions and knowledge translation (Figure 2).

### TPT initiation in priority populations

**FIGURE 3:**
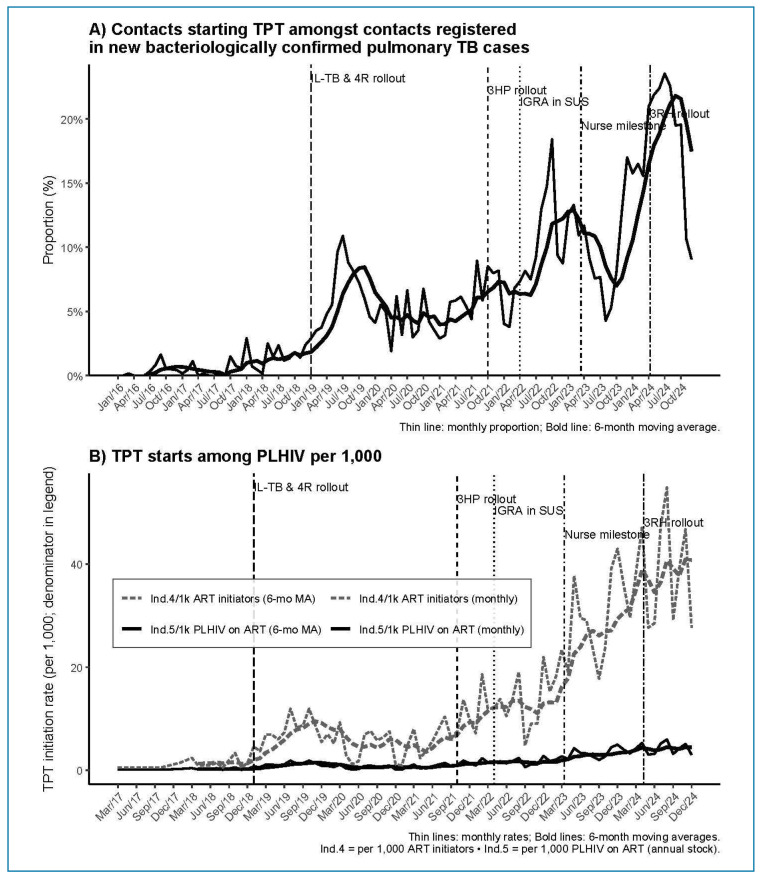
TPT initiation among TB contacts and PLHIV, Amazonas, Brazil, 2016-2024. **Notes: A)** Ratio between TB contacts initiating TPT (IL-TB system) and contacts registered among new bacteriologically confirmed pulmonary TB cases (SINAN-TB system); **B)** TPT initiation rates among PLHIV per 1,000 ART initiators and per 1,000 PLHIV on ART (annual stock), with TPT data from the IL-TB system and ART data from the HIV/AIDS Clinical Monitoring Panel (PIMC); TPT, TB Preventive Treatment; TB, Tuberculosis; 4R, daily doses of rifampicin for four months; 3HP, weekly doses of rifapentine plus isoniazid for three months; IGRA, interferon-gamma release assay; SUS, Brazilian Unified Health System; 3RH, daily doses of rifampicin plus isoniazid for three months; PLHIV, people living with HIV; ART, Antiretroviral Therapy. **Source:** Information System for notification of people undergoing treatment for LTBI (IL-TB) and Notifiable Diseases Information System (SINAN-TB), Amazonas, Brazil; HIV/AIDS Clinical Monitoring Panel, Ministry of Health, Brazil, October 2025.

Among contacts of pulmonary TB cases, the proportion initiating TPT increased steadily, rising from baseline rates below 5% in 2017 to sustained levels between 15 and 23% post-2022. Each programmatic development-IL-TB system, 3HP and IGRA rollout, decentralization of nurses, and introduction of 3RH-corresponded with pronounced jumps in the coverage. Similarly, TPT initiation among PLHIV rose sharply: per 1,000 individuals initiating ART, rates grew from less than 10 to nearly 40 by the end of the study period, especially following nurse-led decentralization and further regimen diversification. These improvements reflect the collective impact of evidence-based strategies, priority targeting, and real-time feedback mechanisms (Figure 3).

### Surveillance, diagnostic access and regimen effectiveness

**FIGURE 4:**
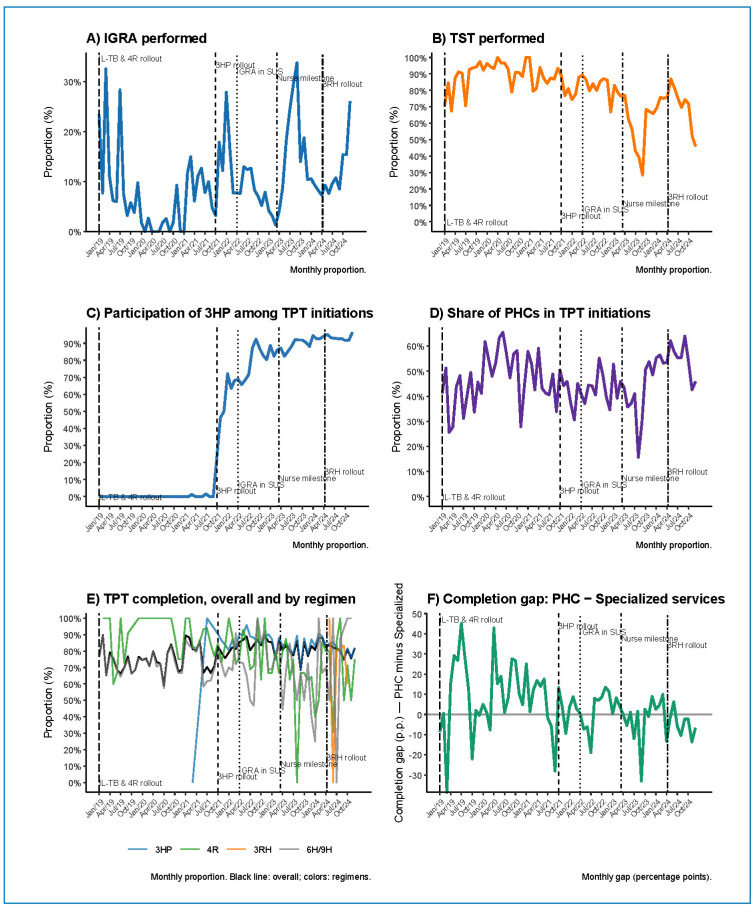
LTBI surveillance indicators, access, and effectiveness of shortened regimens, and TPT, Amazonas, Brazil, Jan 2019-Dec 2024. **Notes: IGRA:** interferon-gamma release assay; **4R:** daily doses of rifampicin for four months; **3HP:** weekly doses of rifapentine plus isoniazid for three months; **SUS:** Brazilian Unified Health System; **3RH:** daily doses of rifampicin plus isoniazid for three months; **TST:** tuberculin skin test; **PHC:** primary health care; **6H/9H:** six or nine months of daily isoniazid. **Source:** Information System for notification of people undergoing treatment for LTBI (IL-TB), Amazonas, Brazil, October 2025.

The proportion of individuals submitted to IGRA for LTBI rose sharply after 2022, peaking at approximately 30% in high-implementation periods, while the use of the traditional TST declined markedly amid shortages of purified protein derivative and diagnostic modernization. Regimen analysis showed a near-complete transition to shorter regimens: within 2 years of the 3HP rollout, the share of TPT initiations using 3HP surpassed 85%. Shifts in care models were evident, with the proportion of primary health care centers participating in TPT rising from less than 30% to more than 60% between 2019 and 2024 in the study area. Completion rates across all regimens highlighted the superior effectiveness of 3HP, which maintained monthly completion rates of over 90%, followed by daily rifampicin for 4 months (4R) and daily rifampicin plus isoniazid for 3 months (3RH), while long-duration regimens of six or nine months of daily isoniazid (6H/9H) achieved only 60-70%. Most notably, the historical completion gap between PHCs and specialized referral services closed steadily over time, eventually favoring PHC delivery, which is a testament to successful nurse-led initiation and decentralization **(** Figure 4 **)**.

## DISCUSSION

The quantitative findings are consistent with global evidence, highlighting persistent gaps in the LTBI cascade of care. Despite the substantial number of contacts reported in the surveillance systems, the proportion initiating TPT remains limited, a pattern widely reported in high-burden settings and emphasized in recent WHO reports and cascade analyses^1^ ^,^ ^2^ ^,^ ^5^. Similarly, although TPT uptake among PLHIV increased over time, the absolute number of individuals initiating preventive therapy remains small relative to the estimated population eligible for treatment, reflecting challenges described in both national and international studies on TB/HIV integration and preventive care delivery^1^ ^,^ ^11^ ^,^ ^31^.

The rapid scale-up and predominance of shortened regimens, particularly 3HP, align with the growing evidence that shorter rifamycin-based regimens are key drivers of improved uptake and completion in programmatic settings^14^ ^,^ ^32^. In parallel, the increasing participation of primary health care and the reduction in differences between levels of care reflect trends observed in implementation studies emphasizing decentralization and task-shifting as critical components for expanding TPT coverage^12^ ^,^ ^33^.

These findings are consistent with prior Brazilian and international studies identifying persistent barriers along the LTBI cascade of care, including health-system constraints, knowledge gaps, and variability in provider engagement^12^ ^,^ ^20^ ^,^ ^22^ ^,^ ^34^.

These results align with implementation studies demonstrating that cascade-informed and context-adapted interventions can substantially increase TPT uptake, as observed in multicountry trials such as ACT4^22^ and CONTACT^35^. In addition, qualitative studies have indicated that patient knowledge of LTBI and effective communication with healthcare providers are key determinants of treatment adherence^36^. In this context, the experience in the state of Amazonas extends this evidence by demonstrating, in a geographically complex real-world setting, that integrating decentralized care, diagnostic innovation, and health system strengthening can further enhance TPT delivery.

Our spatial and temporal analyses of the 3HP rollout confirm prior multicenter evidence that decentralized adoption frequently lags in remote or resource-limited settings and highlight the impact of implementation and logistical support on accelerating uptake^11^ ^,^ ^14^ ^,^ ^22^. This echoes the findings from evaluations of TPT delivery in high-burden regions, as well as the pivotal role of locally generated scientific evidence in informing national policy^7^ ^,^ ^11^ ^,^ ^30^.

In line with implementation studies, IGRA use remained limited in routine practice, while the TST remained the predominant diagnostic method, with variations over time influenced by supply constraints^1^ ^,^ ^6^ ^,^ ^20^ ^,^ ^24^ ^,^ ^25^. The data further validate the superiority of shortened regimens-3HP and 4R-over historical approaches, as reflected in both completion rates and broader population reach^14^ ^,^ ^22^ ^,^ ^32^. The narrowing gap in completion rates between PHCs and specialized services aligns with research advocating for nurse-led, decentralized TPT strategies^33^.

Importantly, ongoing challenges such as losses along the care cascade, especially among contacts and PLHIV, are consistent with analyses from regional and multicenter cohorts^1^ ^,^ ^5^ ^,^ ^7^ ^,^ ^12^ ^,^ ^20^ ^,^ ^21^. These studies have highlighted the importance of workforce training, community engagement, and digital monitoring systems-all themes supported by our surveillance data and the timeline of improvements.

Despite these gains, significant gaps remain in the LTBI care cascade. Although TPT initiation among contacts has increased to 15-23% in recent years, this remains a small proportion of eligible individuals relative to the global TB prevention targets. The WHO End TB Strategy and subsequent commitments call for the large-scale expansion of TPT among household contacts and other high-risk groups, however recent reports indicate that progress remains uneven and insufficient, particularly in high-burden settings^2^. These findings suggest that while programmatic improvements have accelerated TPT uptake, further expansion is needed to achieve meaningful reductions in TB incidence, especially in hard-to-reach and decentralized settings.

Our findings on variability in implementation, persistent geographic disparities^10^ ^-^ ^13^, and periods of supply interruptions^25^ ^,^ ^26^ ^,^ ^37^ ^,^ ^38^ further contribute to the dialogue around program resilience and the capacity for health-system adaptation, as underscored in recent publications addressing both pandemic impacts^1^ ^,^ ^2^ and innovations in TB control^23^ ^,^ ^24^.

These actions were implemented in a context marked by substantial structural challenges, including wide territorial dispersion, low population density, socioeconomic inequalities, and concentration of specialized services in the state capital, as well as the limited availability of qualified professionals for outreach and training in remote municipalities. While some mitigation strategies are reflected in the results, additional operational arrangements played a key role, including coordination among surveillance, health services and academia, decentralized and inter-institutional training approaches, and collaboration across levels of governance to support activities such as expanding TST and strengthening TB-HIV collaborative actions.

The experience in the state of Amazonas underscores the need for continued investment in intersectoral coordination, operational studies, and context-driven policy adaptation-priorities already championed in the Brazilian TB academic and programmatic communities. The sustained engagement of regional scientific centers, including FMT-HVD, and their contributions to multi-center efforts, such as the RePORT-Brazil, have proven critical to both scientific advancement and practical improvement^15^.

Looking forward, our results support the expansion of digital data integration, targeted resource strengthening and systematic evaluation of new models for care delivery and diagnostic management. These priorities are echoed in the literature as essential for meeting the global End TB Strategy goals and closing persistent equity gaps.

This analysis relied on routine aggregated data, which may mask patient-level factors and fail to capture all adverse events or nuances of dropout. The strong representation of the state of Amazonas in multi-center research supports generalizability; however local heterogeneities and the impact of the COVID-19 pandemic must be considered when interpreting the results.

The experience in this high-burden Amazonian setting exemplifies how locally generated evidence and sustained scientific collaboration can effectively strengthen TB prevention programmes, improve equity, and accelerate progress towards the WHO End TB targets. Future priorities should include expanding access to modern diagnostics, strengthening supply chains, leveraging digital innovations and deepening engagement with communities and front-line health care workers. 

The successful expansion of TPT at the regional level represents a compelling intersection of policy, evidence, and practice. Strategic leadership, multi-sectoral collaboration, and rigorous application of locally generated research fundamentally shaped program design and allowed for timely, context-aware responses to both anticipated and emergent challenges. The body of scientific evidence produced in Manaus and regional research centers did not merely inform the local program; it actively drove innovation in diagnostics, adoption of shortened regimens, decentralized care and improved quality across the continuum of TB preventive efforts.

Marked improvements in TPT initiation and completion, particularly among vulnerable communities, were achieved through the flexible integration of research findings into the public health response. The expansion of modern diagnostic tools and nurse-led models, along with continuous monitoring and feedback, has substantially reduced historical gaps in care and positioned this setting as a leading example of TB prevention in high-burden, resource-constrained settings.

Despite these advances, challenges such as geographic equity, resource distribution, and sustained training remain. Future priorities should focus on extending access to innovative tools, reinforcing supply chains, expanding digital data systems and deepening engagement among both communities and frontline health care workers. Ongoing regional research and collaborative learning will remain essential to sustain progress and adapt interventions to meet new needs.

Ultimately, this regional experience underscores that locally led science, evidence-based adaptation and persistent programmatic commitment are prerequisites for achieving the national and global End TB goals. The lessons learned here are not only relevant to Brazil but also offer a blueprint for other settings facing the complex realities of TB prevention and care.

## Data Availability

Research data is available in the body of the article and the data that supports the findings of this study will be available upon reasonable request to the corresponding author of the study.
