# Supplementary material for: Scaling Up Tuberculosis Preventive Treatment in the Brazilian Amazon (2016-2024): a Programmatic Report on Multi-sector Integration in Surveillance, Health Services and Academia
Source: Rev Soc Bras Med Trop. 2026 Jul 17;59(Suppl 1):e0570-2025. doi: 10.1590/0037-8682-0570-2025 (PMC13379189; doi:10.1590/0037-8682-0570-2025)
Supplement: Supplementary material [file 1678-9849-rsbmt-59-s1-e0570-2025-md1.pdf]

SUPPLEMENTARY MATERIAL TABLE 1. Definitions, operationalization, data sources, and limitations of the ten selected indicators used to assess TB preventive treatment implementation in the state of Amazonas, Brazil.

Domain	Indicator	Operational definition	Formula	Numerator/Denominator	Comments/Adjustments	Limitations
Implementation	1. Time to local implementation of 3HP (number of municipalities by time to implementation, months)	Months between Oct/2021 and the first m with ≥1 3HP initiation in the municipality/CNES.	local_implementation_month - Oct/2021 (in months)	—	Sensitivity using thresholds of ≥1 ≥5; useful for diffusion maps.	May overestimate adoption settings with small caseloa
Access	2. TPT initiations among contacts of new bacteriologically confirmed pulmonary TB cases, relative to registered contacts	Proportion of contacts initiating TPT amo registered contacts of notified new bacteriologically confirmed pulmonary TE cases.	contacts_started_period / contacts_registered_period*100	Number of contacts initiating TPT (IL-TB).<br>Number of registered contacts of bacteriologically confirmed pulmo TB cases (SINAN-TB).	Best proxy for contact surveillance performance; consider quality of contact recording.	Underreporting of contacts distort the indicator.
Access (PLI	3. TPT initiations among PLHIV per 1,000 ART initiators	Subanalysis among PLHIV: TPT initiatior relative to the number of individuals initia ART.	(PLHIV_started_TPT / ART_initia * 1,000	PLHIV initiating TPT (IL-TB).<br>Number of PLHIV initiating ART (Ministry of Health HIV Panel).	Requires linkage by municipality/period; interpret as a programmatic proxy for new HIV infection cases.	Coverage and periodicity of data may vary.
Access (PLI	4. TPT initiations among PLHIV per 1,000 PLH currently on ART	Subanalysis among PLHIV: TPT initiatior relative to the annual prevalence of indivi currently on ART (compensates for missi values in ART initiation data).	(PLHIV_started_TPT / PLHIV_on_ART) * 1,000	PLHIV initiating TPT (IL-TB).<br>Number of PLHIV currently on AF annual prevalence estimate (Mini Health HIV Panel).	Useful for long-term monitoring.	Differences in care coverag may confound interpretatior
Diagnosis/ Process	5. Proportion of TPT initiators with IGRA perfor	Proportion of TPT initiators who underwe IGRA.	(IGRA_performed / total_started)	Number of TPT initiators with IGF performed (IL-TB).<br>Total number of TPT initiators (IL-TB).	Stratify pre-2022 vs post-2022; nc concentration in referral services I 2022.	Indication/service bias in th incorporation period.
Diagnosis	6. Proportion of TPT initiators with TST perform	Proportion of TPT initiators in whom a TS was performed.	(TST_performed / total_started) *	Number of TPT initiators with TS recorded in mm (IL-TB).<br>Total number of TPT initiators (IL-TB).	Useful for understanding the TST IGRA transition.	Missing data or reading rec may limit analysis.
Implementat	7. 3HP uptake among TPT initiators	Proportion of TPT initiators receiving the regimen.	(started_3HP / total_started) * 1C	Number of TPT initiators receivin 3HP regimen (IL-TB).<br>Total number of TPT initiators (IL-TB).	Add separate curves for 4R and 3RH.	Short follow-up for 3RH in the recent period.
Implementat Decentraliza	8. Proportion of TPT initiations occurring in PH settings	Proportion of TPT initiations occurring in I facilities.	(started_PHC / total_started) * 1(	Number of TPT initiators in PHC facilities (IL-TB).<br>Total number of TPT initiators (IL-TB).	Assess interaction with the 'nurse prescriber' milestone (from Apr/20 onward).	CNES classification may re cleaning/validation.
Adherence/ Comparisor	9. Difference in TPT completion rates: PHC fac vs. referral hospitals/specialized services	Absolute difference in TPT completion proportions between initiators in PHC fac and those in referral hospitals/specializec services.	%completion_PHC − %completion_specialized_servic (percentage points)	—	Present with 95% CI; adjust by re and period.	Differences in *case-mix* between levels of care.
Adherence/ Effectivenes	10. TPT completion rate (overall cohort and by regimen, excluding individuals with no recordec outcome)	Proportion of individuals in a cohort who completed the TPT regimen.	(completed_cohort / started_coh 100	Number of individuals with record TPT completion (IL-TB).<br>Total number of initiators in the c (IL-TB).	Stratify by regimen (3HP, 4R, 3RH, 6H/9H) and type of service.	Delays in outcome closure underestimate completion i most recent cohorts.

Notes: Indicators were organized across the domains of access, implementation, decentralization, adherence, and diagnostic practices. Numerators and denominators were defined according to the nature of the measure and data source. Proportion-based indicators were derived from routine surveillance data, whereas population-based indicators used annual denominators such as ART stock.

Abbreviations: IL-TB, Information System for notification of people undergoing treatment for LTBI; SINAN-TB, Notifiable Diseases Information System for tuberculosis; PIMC, HIV/AIDS Clinical Monitoring Panel, Ministry of Health, Brazil; TPT, TB preventive treatment; LTBI, latent tuberculosis infection; TB, tuberculosis; PLHIV, people living with HIV; ART, antiretroviral therapy; PHC, primary health care; IGRA, interferon-gamma release assay; TST, tuberculin skin test; CI, confidence interval; CNES, National Registry of Health Establishments; 6H/9H, daily doses of isoniazid for six or nine months; 4R, daily doses of rifampicin for four months; 3HP, weekly doses of rifapentine plus isoniazid for three months; 3RH, daily doses of rifampicin plus isoniazid for three months.

Source: Developed by the authors based on IL-TB and SINAN-TB systems, the HIV/AIDS Clinical Monitoring Panel, Ministry of Health technical documents, and programmatic records from the State Tuberculosis Programme of Amazonas, Brazil.
