# Supplementary material for: Scaling Up Tuberculosis Preventive Treatment in the Brazilian Amazon (2016-2024): a Programmatic Report on Multi-sector Integration in Surveillance, Health Services and Academia
Source: Rev Soc Bras Med Trop. 2026 Jul 17;59(Suppl 1):e0570-2025. doi: 10.1590/0037-8682-0570-2025 (PMC13379189; doi:10.1590/0037-8682-0570-2025)
Supplement: Supplementary material [file 1678-9849-rsbmt-59-s1-e0570-2025-md2.pdf]

SUPPLEMENTARY MATERIAL TABLE 2. Annual results of selected indicators for TB preventive treatment implementation in Amazonas, Brazil,2016–2024.

Indicator	Calculation Method / Description / Data Source	Annual Results by Year and Summary Statistical Measures								
		2016	2017	2018	2019	2020	2021	2022	2023	2024
1. Time to local implementation of 3HP (number of municipalities by time to implementation, months)	Difference in months between October 2021 and the first month with ≥1 TPT initiations with 3HP in the municipality (IL-TB)	<3 months	3 to 5 months	6 to 11 months	12 to 23 months	24 to 36 months		Median	Interquartile Range	
		1	1	11	4	10		12	8 – 28.5	
2. TPT initiations among contacts of new bacteriologically confirmed pulmonary TB cases, relative to registered contacts	Number of contacts initiating TPT (IL-TB)	23	29	125	547	325	499	841	945	1667
	Number of registered contacts of new bacteriologically confirmed pulmonary TB cases (SINAN-TB)	6559	7150	8146	8853	7365	8087	9059	9596	9043
	Result (%)	0,4%	0,4%	1,5%	6,2%	4,4%	6,2%	9,3%	9,8%	18,4%
	95% CI	0.23 – 0.53	0.28 – 0.58	1.29 – 1.83	5.70 – 6.70	3.97 – 4.91	5.67 – 6.72	8.70 – 9.90	9.27 – 10.46	17.65 – 19.25
3. TPT initiations among PLHIV per 1,000 ART initiators	Number of PLHIV initiating TPT (IL-TB)	-	2	31	197	108	173	318	660	959
	Number of PLHIV initiating ART (Ministry of Health HIV Panel)	-	1903	2096	2181	1724	2123	2098	2095	2173
	Result (per 1,000)	-	1,05	14,79	90,33	62,65	81,49	151,57	315,04	441,33
	95% CI	-	0.13 – 3.80	10.05 – 20.99	78.15 – 103.86	51.39 – 75.63	69.80 – 94.58	135.37 – 169.18	291.46 – 340.01	413.83 – 470.17
4. TPT initiations among PLHIV per 1,000 PLHIV currently on ART	Number of PLHIV initiating TPT (IL-TB)	-	-	-	197	108	173	318	660	959
	Number of PLHIV currently on ART – annual prevalence estimate (Ministry of Health HIV Panel)	-	-	-	13879	14556	15651	16661	18185	19510
	Result (per 1,000)	-	-	-	14,19	7,42	11,05	19,09	36,29	49,15
	95% CI	-	-	-	12.28 – 16.32	6.09 – 8.96	9.47 – 12.83	17.05 – 21.30	33.58 – 39.17	46.09 – 52.37
5. Proportion of TPT initiators with IGRA performed	Number of TPT initiators with IGRA performed (IL-TB)	23	30	77	97	10	83	139	227	276
	Total number of TPT initiators (IL-TB)	23	30	150	865	525	821	1379	1756	2571
	Result (%)	100,0	100,0	51,3	11,2	1,9	10,1	10,1	12,9	10,7
	95% CI	85.7 – 100.0	88.6 – 100.0	43.4 – 59.2	9.3 – 13.5	1.0 – 3.5	8.2 – 12.4	8.6 – 11.8	11.4 – 14.6	9.6 – 12.0
6. Proportion of TPT initiators with TST performed	Number of TPT initiators with TST performed (IL-TB)	0	0	71	755	487	705	1137	1129	1877
	Total number of TPT initiators (IL-TB)	23	30	150	865	525	821	1379	1756	2571
	Result (%)	0,0	0,0	47,3	87,3	92,8	85,9	82,5	64,3	73,0
	95% CI	0.0 – 14.3	0.0 – 11.4	39.5 – 55.3	84.9 – 89.3	90.2 – 94.7	83.3 – 88.1	80.4 – 84.4	62.0 – 66.5	71.3 – 74.7
7. 3HP uptake among TPT initiators	Number of TPT initiators receiving the 3HP regimen (IL-TB)	-	-	-	-	-	104	1073	1538	2399
	Total number of TPT initiators (IL-TB)	-	-	-	-	-	821	1379	1756	2571
	Result (%)	-	-	-	-	-	12,7	77,8	87,6	93,3
	95% CI	-	-	-	-	-	10.6 – 15.1	75.5 – 79.9	86.0 – 89.0	92.3 – 94.2
8. Proportion of TPT initiations occurring in PHC settings	Number of TPT initiators in PHC facilities (IL-TB)	0	0	29	345	278	377	585	750	1.419
	Total number of TPT initiators (IL-TB)	23	30	150	865	525	821	1379	1756	2571
	Result (%)	0,0	0,0	19,3	39,9	53,0	45,9	42,4	42,7	55,2
	95% CI	0.0 – 14.3	0.0 – 11.4	13.8 – 26.4	36.7 – 43.2	48.7 – 57.2	42.5 – 49.3	39.8 – 45.0	40.4 – 45.0	53.3 – 57.1
9. Difference in TPT completion rates: PHC facilities vs. referral hospitals/specialized services	TPT completion rate (%) among initiators in PHC facilities	-	-	75,9	80,0	77,3	79,8	86,7	81,1	80,5
	TPT completion rate (%) among initiators in referral hospitals and specialized TB services	-	-	61,2	66,9	67,6	75,0	83,5	81,4	83,4
	Difference (percentage points)	-	-	+14.7	+13.1	+9.7	+4.8	+3.2	-0.3	-2.9
10. TPT completion rate (overall cohort and by regimen, excluding individuals with no recorded outcome)	10.1. Number of individuals with recorded TPT completion (IL-TB)	16	25	96	624	382	634	1.170	1.417	2.046
	Total number of initiators in the cohort (IL-TB)	23	29	150	865	525	821	1.379	1.744	2.501
	Result (%)	69,6	86,2	64,0	72,1	72,8	77,2	84,8	81,3	81,8
	95% CI	49.1 – 84.4	69.4 – 94.5	56.1 – 71.2	69.1 – 75.0	68.8 – 76.4	74.2 – 80.0	82.9 – 86.6	79.4 – 83.0	80.2 – 83.3
	10.2. Number of individuals with recorded TPT completion on the 6H/9H regimen (IL-TB)	16	25	96	586	356	385	127	87	31
	Number of 6H/9H regimen initiators in the cohort (IL-TB)	23	29	150	820	495	529	180	128	46
	Result (%)	69,6	86,2	64,0	71,5	71,9	72,8	70,6	68,0	67,4
	95% CI	49.1 – 84.4	69.4 – 94.5	56.1 – 71.2	68.3 – 74.4	67.8 – 75.7	68.8 – 76.4	63.5 – 76.7	59.5 – 75.4	53.0 – 79.1
	10.3. Number of individuals with recorded TPT completion on the 4R regimen (IL-TB)	-	-	-	38	26	160	101	62	46
	Number of 4R regimen initiators in the cohort (IL-TB)	-	-	-	45	30	188	126	87	74
	Result (%)	-	-	-	84,4	86,7	85,1	80,2	71,3	62,2
	95% CI	-	-	-	71.2 – 92.3	70.3 – 94.7	79.3 – 89.5	72.3 – 86.2	61.0 – 79.7	50.8 – 72.4
	10.4. Number of individuals with recorded TPT completion on the 3HP regimen (IL-TB)	-	-	-	-	-	89	942	1.268	1.955
	Number of 3HP regimen initiators in the cohort (IL-TB)	-	-	-	-	-	104	1.073	1.529	2.362
	Result (%)	-	-	-	-	-	85,6	87,8	82,9	82,8
	95% CI	-	-	-	-	-	77.6 – 91.1	85.7 – 89.6	81.0 – 84.7	81.2 – 84.2
	10.5. Number of individuals with recorded TPT completion on other/unspecified regimens (IL-TB)	-	-	-	-	-	-	-	-	14
	Number of other/unspecified regimen initiators in the cohort (IL-TB)	-	-	-	-	-	-	-	-	19
	Result (%)	-	-	-	-	-	-	-	-	73,7
	95% CI	-	-	-	-	-	-	-	-	51.2 – 88.2

Notes: TPT, TB preventive treatment; TB, tuberculosis; PLHIV, people living with HIV; ART, antiretroviral therapy; PHC, primary health care; IGRA, interferon-gamma release assay; TST, tuberculin skin test; 3HP, weekly rifapentine plus isoniazid for 3 months; 4R, daily rifampicin for 4 months; 3RH, daily rifampicin plus isoniazid for three months; 6H or 9H, six or nine months of daily isoniazid; IQR, interquartile range; CI, confidence interval. Proportions are presented with 95% confidence intervals estimated using the Wilson's method for binomial distributions; rates per 1,000 are presented with 95% confidence intervals estimated using exact Poisson method. Absolute differences are expressed in percentage points. Annual estimates were calculated according to the measure of each indicator and denominator data source.

Source: Information System for notification of people undergoing treatment for LTBI (IL-TB) and Notifiable Diseases Information System (SINAN-TB), Amazonas, Brazil; HIV/AIDS Clinical Monitoring Panel, Ministry of Health, Brazil, October 2025.
